# Effect of Siguan Acupuncture on Gastrointestinal Motility: A Randomized, Sham-Controlled, Crossover Trial

**DOI:** 10.1155/2013/918392

**Published:** 2013-05-16

**Authors:** Kyung-Min Shin, Ji-Eun Park, Sanghun Lee, Sun-Mi Choi, Yo-Chan Ahn, Jin-Woo Lee, Jin-Hee Kim, Chang-Gue Son

**Affiliations:** ^1^Department of Medical Research, Korea Institute of Oriental Medicine, Daejeon, Republic of Korea; ^2^Department of Health Service Management, Daejeon University, Daejeon, Republic of Korea; ^3^Department of Radiology, Oriental Hospital of Daejeon University, Daejeon, Republic of Korea; ^4^Liver and Immunology Research Center, Oriental Hospital of Daejeon University, 22-5 Daejung-dong, Jung-gu, Daejeon 301-724, Republic of Korea

## Abstract

Siguan acupoints have been used to treat gastrointestinal symptoms in acupuncture practices for a long time. This study aimed to investigate the effects of Siguan acupuncture on gastrointestinal motility under accelerated conditions using a randomized, sham-acupuncture-controlled, crossover study. Twenty-one healthy male subjects were hospitalized and randomized into either a real acupuncture group (at Siguan acupoints) or a sham acupuncture group. Subjects were administered with mosapride citrate (15 mg a day) for 2 days starting 24 hours before the first acupuncture treatment. Immediately after the administration of radio markers, acupuncture treatment was conducted 4 times at 12-hour intervals. Gastrointestinal motility was assessed using radiograph distribution of the radio-markers located in the small intestine, ascending colon, transverse colon, descending colon, rectum, and outside the body immediately after the first acupuncture treatment and at 6, 12, 24, and 48 hours. After a 2-week washout period, the real acupuncture group in the first session was treated with sham acupuncture in the second session, and vice versa. Gastrointestinal motility was generally reduced in the real acupuncture group compared with the sham acupuncture group throughout the 4 different time points. A significant difference was observed at 24 hours following the first acupuncture treatment (*P* < 0.05).

## 1. Introduction

Acupuncture is a typical therapy applied in traditional Chinese and Korean medicine that stimulates specific acupoints on the human body either manually or electronically. The medical application rates of acupuncture have steadily increased for various conditions, including pain, obesity, stroke rehabilitation, gastrointestinal disorders, psychological illnesses, and metabolic diseases [1].

The effects of acupuncture treatment may depend on the selected acupoints, the combination of acupoints, and the intensity of stimulation. Siguan acupoints (a combination of bilateral LI4 and LR3, meaning “*four gates*” in Chinese) are the most commonly used acupoints for various symptoms, especially gastrointestinal (GI) symptoms such as constipation and diarrhea [2, 3].

Traditional Chinese medicine stresses that acupuncture is applied to restore a balance between “ying” and “yang” and to regulate the flow of “qi” and “blood.” The mechanisms of acupuncture are unclear; however, many studies have reported that acupuncture treatment contributes to the maintenance of the biochemical balance of the central nervous system [4, 5]. According to traditional meridian theory, acupuncture functions via homeostatic mechanisms [6]. Therefore, acupuncture performed at the same acupoints can be used to treat the opposing symptoms. For example, acupuncture performed at GV20 has been shown to be effective in the treatment of hypertension [7] and hypotension [8]. Additionally, the same stimuli performed at the same acupoints induce different responses depending on the physiological or pathological state of the body. Acupuncture performed at LI4 and PC6 reduces heart rate variability when the patient is in a state of fatigue, but it has no effect when the individual is in a normal state [9].

Based upon the previous observations, we hypothesized that Siguan acupuncture affects GI motility as normalizing manners depend on normal, suppressed and excessive status. We previously showed that Siguan acupuncture does not influence GI motility in healthy subjects [10], while it accelerates GI motility in individuals in a loperamide-induced suppressed condition [11].

As a serial study, we conducted an additional trial to understand whether Siguan acupuncture reduces the activity of GI motility under mosapride citrate-induced excessive condition.

## 2. Materials and Methods

### 2.1. Subjects

Twenty-three healthy male subjects were recruited in this study. Only those who had normal finding of complete blood count, liver function test, urinary test, and radiographic investigation (simple abdomen) and were negative for human immunodeficiency virus (HIV), hepatitis B virus (HBV), and hepatitis C virus (HCV) were allowed to participate. Subjects were excluded from the study if they had symptoms of constipation or diarrhea, a diagnosis of irritable bowel syndrome or functional constipation, inflammatory bowel disease or other structural bowel diseases, or other significant disorders or diseases that could interfere with the completion of the study. Participants who smoked or drank alcohol 3 days prior to trial inclusion were also excluded. Finally, 21 healthy subjects (median age: 22 years, range: 19–25 years; median height: 173 cm, range: 165–183 cm; and median weight: 71 kg, range: 59–88 kg) were enrolled in this study (Table 1).

The study was conducted in accordance with the Declaration of Helsinki, and written informed consent was obtained from each participant prior to study enrollment. The study was approved by the Institutional Review Board of Daejeon University Hospital, Daejeon, Republic of Korea (Authorization number DJOMC-89).

### 2.2. Study Design

**Figure 1:**
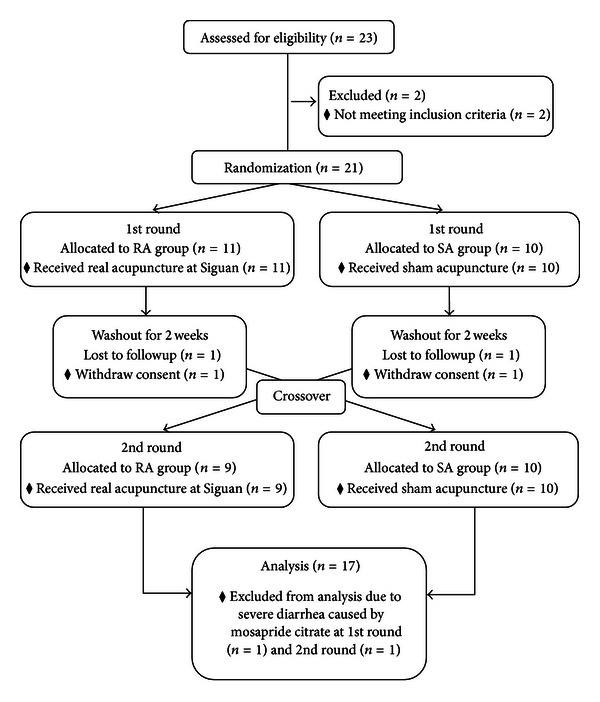
CONSORT flow chart.

This study was a single-blind, randomized, crossover, sham-acupuncture-controlled clinical trial performed to evaluate the effect of Siguan acupuncture under mosapride citrate-induced excessive conditions of GI motility. A Korean Medical Doctor (KMD) initially screened each potential participant against the inclusion and exclusion criteria. Next, eligible participants were randomized in a crossover manner into either a real acupuncture group (RA group, acupunctured at Siguan points) or a sham acupuncture group (SA group, minimal-acupuncture applied at nonacupoints). A 2-week washout period was included between the two sessions; the RA group in the first session was treated with SA in the second session, and vice versa (Figure 1).

**Figure 2:**
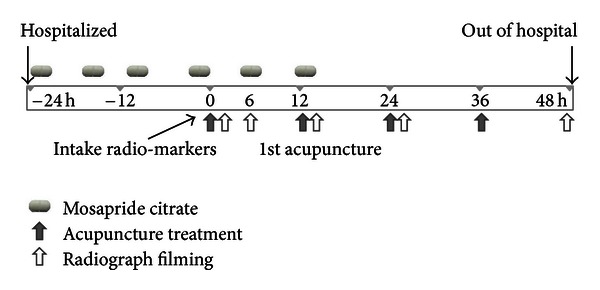
Study scheme. Subjects were administered 15 mg of mosapride citrate per day (3 treatments of 5 mg) for 2 days starting 24 hours before the first acupuncture treatment. Immediately after the administration of radio markers, acupuncture treatment was conducted at 12-hour intervals. Gastrointestinal motility was measured via radiography immediately after the first acupuncture treatment and at 6, 12, 24, and 48 hours thereafter.

All subjects were hospitalized, and then an excessive condition of GI motility was induced using a GI movement modifier. All subjects were administered 15 mg of mosapride citrate (divided three, Gasmotin Tab. Daewoong Pharmaceutical Co. Ltd, Seoul, Republic of Korea) per day starting from the day prior to the first acupuncture treatment and continuing for 2 days. For assessment of GI motility, all participants were administered radio markers (Kolomaker TM; GAIA Medical Corporation, Seoul, Republic of Korea) immediately before the first acupuncture treatment, and GI motility was measured via radiography immediately after the first acupuncture treatment and at 6, 12, 24, and 48 hours thereafter (Figure 2). Additionally, the frequency of defecation was monitored starting from 1 day before the acupuncture treatment to 2 days thereafter. Blood pressure was monitored once a day, and adverse events were assessed.

### 2.3. Acupuncture Treatment

**Figure 3:**
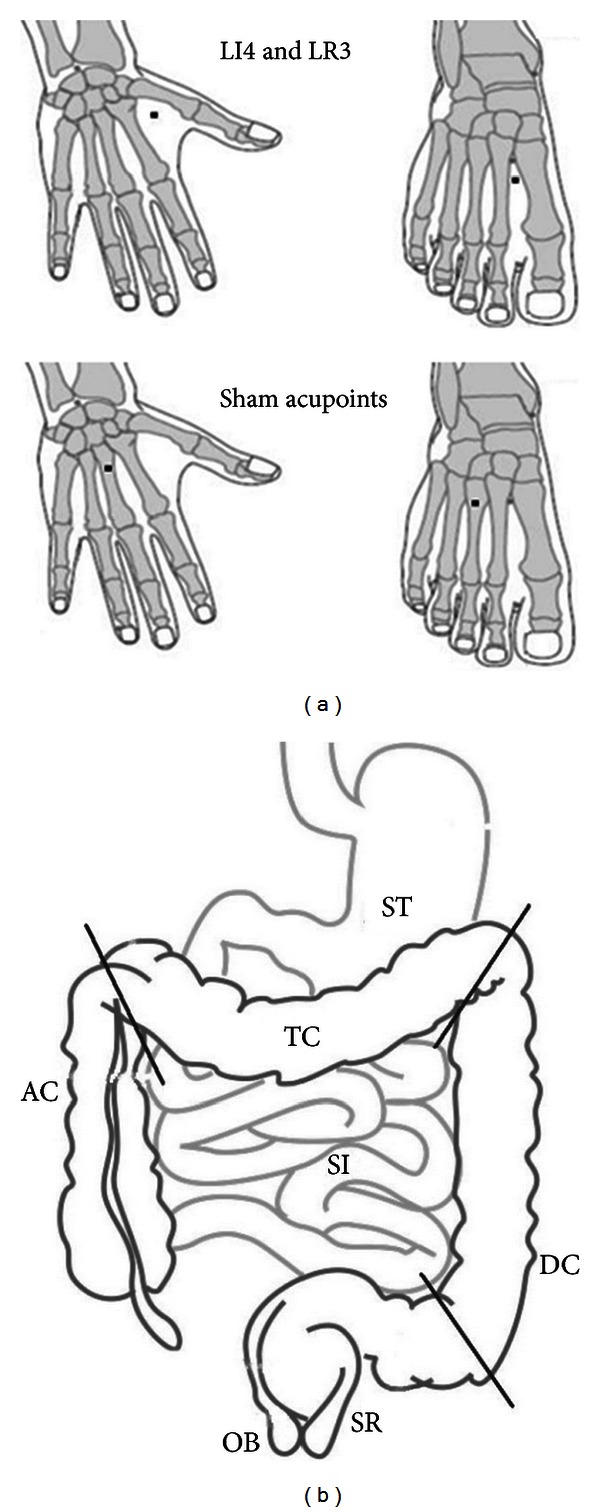
Acupoints and illustration of the alimentary canal. (a) Siguan acupoints (bilateral LI4 and LR3) and sham acupoints. The sham acupoints were applied to the nonacupoints 2-3 cm laterally from LI4 and LR3 on both hands and feet. (b) A simple illustration of the alimentary canal. Abbreviations: ST, stomach; SI, small intestine; AC, ascending colon; TC, transverse colon; DC, descending colon; SR, sigmoid/rectum; and OB, outside body.

For the RA group, the needle was inserted perpendicularly at an approximate depth of 1 cm at bilateral LI4 and LR3 and rotated 90 degrees 5 times to provoke a *De-qi* sensation in the sequential order as follows: right hand, left hand, right foot, and left foot. For the SA group, the needle was inserted at a depth of 0.3–0.5 cm (transversely toward the lateral side), and no manipulation of the needle was performed to avoid inducing *De-qi*. The size of the acupuncture needle (gauge, 30; diameter, 0.30 mm; Dongbang Healthcare Products, Seoul, Republic of Korea), retaining time (20 minutes), and treatment frequencies were consistent between the two groups. The acupuncture treatment was conducted 4 times at 12-hour intervals. In the SA group, a sham point of LI4 was localized at the middle of the junction of the capitate bone and the third metacarpal bone, while a sham point of LR3 was localized at the middle of the junction of the lateral cuneiform bone and the third metatarsal bone (Figure 3(a)). The practitioner was acupuncture specialist who has had 20 years of clinical experience in oriental internal medicine.

### 2.4. Radiological Analysis

GI motility was analyzed using the exponential weighted score (EWS) method [12]; the presence of each radio marker located in the small intestine, ascending colon, transverse colon, descending colon, rectum, and outside the body was scored as 1, 2, 3, 4, 5, and 6, respectively (Figure 3(b)). The total scores of GI motility were calculated according to the distributions of 20 radio markers at each time point. One specialist in diagnostic radiology, who was blinded to the randomization, analyzed the radiographs.

### 2.5. Statistical Analysis

All data were entered into a data sheet twice and reviewed to ensure accuracy. A per-protocol (PP) analysis was conducted. All data were summarized as the mean ± standard deviation for continuous data and as the frequency (%) for dichotomous data. A Student's *t*-test was used to analyze the differences of the mean change in scores between the two groups at 6, 12, 24, and 48 hours. Statistical analysis was performed using the SAS statistical package (v.9.1; SAS institute Inc., Cary, NC, USA), and the level of significance was established at *P* = 0.05.

## 3. Results 

### 3.1. Effects on Gastrointestinal Motility

Along with the time points of radiography, both the RA and SA groups showed an increase in EWS values, thereby indicating the gradual passage of radio markers from the stomach and small intestine into the rectum and outside of the body. Almost no radioactivity was observed in the radiographs of either the RA or SA groups after 48 hours of radio markers administration, which indicates that most radio markers had already been defecated. For all radiographies, the EWS values were lower in the RA group compared with the SA group. In particular, the differences in the value changes between the two groups were significant at 24 hours after the first acupuncture treatment (*P* < 0.05). At 6, 12, and 48 hours after radio markers intake, no significant difference was observed in GI motility (*P* > 0.05, Table 2).

### 3.2. Effect on Defecation Frequency

Because of the relationship between intestinal movement and defecation, the number of times each subject defecated was accurately recorded before and during the trial. The mean frequency of daily defecations for all subjects was 0.9 ± 0.4 before the trial. The administration of mosapride citrate slightly increased the frequency of daily defecations by approximately 1.3 times. No significant change in the average number of defecations between the two groups was observed (Table 3).

### 3.3. Successful Completion Rates and Adverse Effects

Two subjects withdrew from the study due to personal reasons after the first session. Nineteen subjects completed the two sessions of procedures. Two subjects reported severe diarrhea during the first session (RA group) and the second session (SA group); therefore, these individuals were excluded from the final data analysis. No acupuncture-associated adverse events or abnormal blood pressure measurements were reported during the two sessions of the trial.

## 4. Discussion

Acupuncture originated in east-Asia during ancient times and is now used for the treatment of various conditions worldwide [13]. Numerous studies have demonstrated the therapeutic effects of acupuncture for a variety of conditions, such as chronic pain (back and neck pain, osteoarthritis, chronic headache, and shoulder pain) [14], osteoarthritis [15–17], dental pain [18] as well as nausea, and vomiting [19, 20]. Moreover, acupuncture is a relatively safe treatment, although minor adverse events, including feelings of faintness and syncope, have been rarely observed following acupuncture treatment [21, 22].

Siguan acupuncture has been used for various conditions, including respiratory failure [23], chronic fatigue syndrome [24], primary dysmenorrheal [24], and particularly disorders of the digestive system [2, 3]. Distortion of GI motility can lead to GI symptoms such as abdominal pain, constipation, and diarrhea. Siguan acupuncture is also believed to be partially associated with the autonomic nervous system. In animal study using rats, acupuncture stimulation to the acupoints on forelimbs and hindlimbs affected GI motility via modulation of vagus nerves and sympathetic nerves [25]. Therefore, the therapeutic mechanisms of Siguan acupuncture on the GI motility are thought to modulate GI motility.

Based on our series of studies, we investigated the hypothesis that Siguan acupuncture acts by modulating GI motility differently depending on the status of its activities. A prior study was conducted on normal adults for whom no effect on GI motility was observed with Siguan acupuncture [10], while Siguan acupuncture improved suppressed GI motility with loperamide [11]. In the current study, we investigated whether Siguan acupuncture suppresses the activity of GI motility under GI hypermotility conditions.

GI motility varies between individuals [26] and is closely associated with food [27], stress [28], and psychological states such as anxiety and depression [29]. Accordingly, this study was conducted with a crossover design to overcome the individual differences of basal levels of transit time for the entire gut. Additionally, all the subjects were hospitalized to ensure that they would have the same meals and life style patterns throughout the two sessions. The GI motility is easily affected by menstruation-related emotion and pain; therefore, we recruited only a male in this study. To generate an excessive condition of GI motility, all subjects were administered mosapride citrate for 2 days. Mosapride citrate is a serotonin 5-hydroxytryptan-4 (5-HT_4_) receptor agonist that enhances the gastric accommodation reflex and antral contractions, and increases gastric motility and gastric emptying [30–32]. Comparing the average frequency of defecation (0.9 ± 0.4 times per day), the defecation frequency was increased approximately 1.3 times.

To evaluate the overall effects of Siguan acupuncture, we examined the distribution of 20 radio markers assigned a weighted score according to the passage from the stomach at 4 time points. As expected, the EWS scores increased with time following the intake of radio markers. The pattern of GI motility was generally lower in the RA group than in the SA group throughout the experimental period. The greatest difference was observed at 24 hours following the first acupuncture treatment, which was statistically significant (*P* < 0.05). At 6, 12, and 48 hours after the administration of radio markers, no significant difference was observed in GI motility (*P* > 0.05). These results show that Siguan acupuncture lowers GI motility under conditions of a mosapride citrate-induced acceleration of GI motility. And to present the statistical significance especially at 24 hours would be associated with that radio-markers' distribution had passed GI track by moderated distance where is optimal compared with other time-points. This finding supports the clinical observations that Siguan acupuncture displays therapeutic effects for various disorders, including diarrhea and constipation. One study showed that the change in GI motility was observed at a stimulation level of acupuncture exceeding the threshold for A*δ* and/or C afferent fiber activation [25]. Therefore, we expect that a stronger stimulation at Siguan, such as electroacupuncture, would induce a more significant modification of GI motility.

Two participants withdrew consent after the first session, and two participants were excluded from the data analysis because they tested negative for radio markers due to severe diarrhea. The subjects who completed the two sessions (*n* = 17) were included in the statistical analysis. Although we adapted a crossover design, this study has several limitations. First, the assessment of GI motility is restraint, as the EWS score for both groups reaches the maximum score when total radio markers pass outside of the body. Second, this study was conducted on an artificially inducted pathology, which may vary in the real status. In fact, GI hypermotility was lower than we expected by 1.5 times, and two participants had diarrhea.

The main purpose of acupuncture is to balance and normalize body functions in traditional Meridian theory. Our serial studies strongly suggest that Siguan acupuncture tends to normalize the abnormal state of GI motility. We propose that our results are evidence for the general mechanism of acupuncture action. Further studies are required to confirm the effects of Siguan acupuncture in patients with various states of GI motility, such as constipation, diarrhea, and irritable bowel syndrome.

## 5. Conclusions

This study partially evidenced that Siguan acupuncture can modulate GI motility a therapeutic manner under mosapride citrate-induced excessive condition.

## Figures and Tables

**Table 1:** Demographics of study participants at baseline.

	Mean ± SD (*n* = 21)	Minimum	Maximum
Age (year)	22.0 ± 1.9	19	25
Height (cm)	173.0 ± 34.3	165	183
Weight (kg)	71.0 ± 8.0	59	88
Body temperature (°C)	36.6 ± 0.2	36	37
Blood pressure (mmHg)			
Systolic	120.0 ± 8.7	100	130
Diastolic	80.0 ± 6.0	70	90
Pulse (beats/min)	74.0 ± 5.8	58	82

Results are reported as mean ± standard deviation (SD).

**Table 2:** Change in exponential weighted score at four different time points.

Time point	Group	Exponential weighted score	*t* value	*P* value	95% confidence interval
6 h	RA group	12.8 ± 10.9	0.458	0.653	(7.6, 18.0)
	SA group	14.1 ± 9.80			(9.4, 18.8)
12 h	RA group	43.5 ± 16.2	1.112	0.282	(35.8, 51.2)
	SA group	49.6 ± 22.8			(38.7, 60.4)
24 h	RA group	66.9 ± 20.0	2.220	0.041*	(57.5, 76.4)
	SA group	76.1 ± 17.1			(68.0, 84.3)
48 h	RA group	92.1 ± 13.5	1.258	0.226	(85.7, 98.5)
	SA group	96.1 ± 7.70			(92.4, 99.7)

The results show altered values of the weighted score for radio-markers movement in the RA and SA groups at four different time points compared with time 0. The results are expressed as the mean ± standard deviation.

*Represents a significant difference between the two groups with *P *< 0.05 as assessed via Student's *t*-test.

**Table 3:** Frequency of defecation during the treatments.

Time point	Group	Frequency of defecation (times/day)	*t* value	*P* value	95% confidence interval
Before trial	Total subjects	0.9 ± 0.4	—	—	—
24 h	RA group	1.3 ± 0.9	−0.477	0.637	(0.9, 1.7)
	SA group	1.1 ± 1.3			(0.5, 1.7)
48 h	RA group	1.3 ± 0.9	0.000	1.000	(0.9, 1.7)
	SA group	1.3 ± 0.9			(0.9, 1.7)

The results show the defecation frequency of the RA and SA groups at three different time points. No statistical significance was observed between the two groups.
